# Coarse-grained molecular dynamics studies of the translocation mechanism of polyarginines across asymmetric membrane under tension

**DOI:** 10.1038/srep12808

**Published:** 2015-08-03

**Authors:** XiaoCong He, Min Lin, BaoYong Sha, ShangSheng Feng, XingHua Shi, ZhiGuo Qu, Feng Xu

**Affiliations:** 1 Key Laboratory of Thermo-Fluid Science and Engineering of Ministry of Education, School of Energy and Power Engineering, Xi’an Jiaotong University, Xi’an 710049, P.R. China; 2 Bioinspired Engineering and Biomechanics Center (BEBC), Xi’an Jiaotong University, Xi’an 710049, P.R. China; 3 The Key Laboratory of Biomedical Information Engineering of Ministry of Education, School of Life Science and Technology, Xi’an Jiaotong University, Xi’an 710049, P.R. China; 4 Institute of Basic Medical Science, Xi’an Medical University, Xi’an 710021, P.R. China; 5 The State Key Laboratory of Nonlinear Mechanics, Institute of Mechanics, Chinese Academy of Sciences.

## Abstract

Understanding interactions between cell-penetrating peptides and biomembrane under tension can help improve drug delivery and elucidate mechanisms underlying fundamental cellular events. As far as the effect of membrane tension on translocation, it is generally thought that tension should disorder the membrane structure and weaken its strength, thereby facilitating penetration. However, our coarse-grained molecular dynamics simulation results showed that membrane tension can restrain polyarginine translocation across the asymmetric membrane and that this effect increases with increasing membrane tension. We also analyzed the structural properties and lipid topology of the tensed membrane to explain the phenomena. Simulation results provide important molecular information on the potential translocation mechanism of peptides across the asymmetric membrane under tension as well as new insights in drug and gene delivery.

Cell-penetrating peptides (CPPs) are short sequences of amino acids that have recently attracted significant attention as drug carriers because of their ability to cross cell membranes alone or with cargo (e.g., DNA, proteins, or other particles)^1^,^2^,^3^,^4^. For instance, tans-activator of transcription (TAT) peptide and mesoporous silica nanoparticle conjugates can deliver the anticancer drug doxorubicin into targeted nucleus with high efficiency^5^. Complexes of amphipathic peptide and plasmid deoxyribonucleic acid can cross the cytomembrane of cancer and fibroblast cells^6^. Understanding CPP–biomembrane interactions is crucial in elucidating the potential mechanisms underlying fundamental cellular events and subsequent therapeutics. Tremendous efforts have been exerted to explore the translocation mechanism of CPPs, which may include endocytosis^7^ and direct penetration^8^,^9^. While arginine-rich peptides can be internalized by cells through macropinocytic uptake, the peptides can also pass through the membrane through direct translocation when the macropinocytic pathway is inhibited^7^. Therefore, the exact pathway through which CPPs enter cells remains incompletely understood.

As a selectively permeable barrier to ions and organic molecules in all living organisms, the membrane plays an important role in CPP–biomembrane interactions^10^. In many cases, osmotic pressure and external forces (e.g., cells located in blood vessels and lungs experience dilation forces over large areas) can stretch the membrane and position it under tension^11^,^12^. Membrane tension can organize the shape and motility of cells and regulate cell behaviors such as membrane trafficking and cell protrusion^13^. Tensed membranes can influence the permeability of chemotherapeutic agents^14^,^15^ and induce nanoparticle toxicity^16^. However, little is known about the effect of membrane tension on CPP translocation. Therefore, understanding the detailed influence of tensed membranes on CPP–biomembrane interactions is important.

Biomembranes are generally considered as symmetric, but the lipid distribution in most eukaryotic cell membranes is asymmetric. For instance, human erythrocyte membrane comprises phosphatidylcholine, sphingomyelin, and glycolipids in the outer leaflet and phosphatidylethanolamine, phosphatidylserine, and phosphatidylinositol in the inner leaflet^10^,^17^,^18^. Such membrane asymmetry can affect biological behaviors, such as recognition by macrophages^19^ and cellular uptake^20^. Several simulation studies have considered the asymmetric membrane for NP–biomembrane interactions, which include the scenario that dipalmitoyl phosphatidylserine (DPPS) lipids in the inner membrane leaflet can change the equilibrated location of benzocaine in the membrane^21^ and enhance poly amidoamine (PAMAM) dendrimer penetration through the membrane^20^. The potential of mean force (PMF) curve is asymmetric in interactions between peptides and the asymmetric membrane which indicates that the state of peptides adsorbed onto the inner membrane leaflet is stable^22^. Therefore, the effect of membrane asymmetry on nanoparticle–biomembrane interactions must be considered.

Among the various CPPs identified thus far, polyarginine peptides (R8) are particularly interesting because of their higher translocation efficiency compared with other CPPs (e.g., polylysine and TAT peptides)^21^. To investigate the influence of membrane tension on the R8 peptide translocation mechanism, we performed coarse-grained molecular dynamics (CGMD) simulations based on the polyarginine peptides and asymmetric lipid bilayer model. The effects of R8 peptide concentrations and original distributions are also discussed, and tensed membrane properties are analyzed. Our study provides insights into the interactions between CPPs and tensed biomembranes at the molecular level as well as suggestions for therapeutic purposes, such as gene and targeted drug delivery.

## Model and Methods

CGMD simulation is a powerful tool for studying CPP–biomembrane interactions that presents a larger system and longer simulation time than traditional all-atom simulations^22^,^23^,^24^. As a typical coarse-grained force field, the Martini coarse-grained force field can simulate biological systems, including membranes, proteins, and genes^25^. This coarse-grained (CG) force field defines four particle types, namely, polar (P), apolar (C), nonpolar (N), and charged (Q). In general, each CG particle represents four heavy atoms; thus, the effective time is four times faster than the atomistic model^26^.

**Figure 1:**
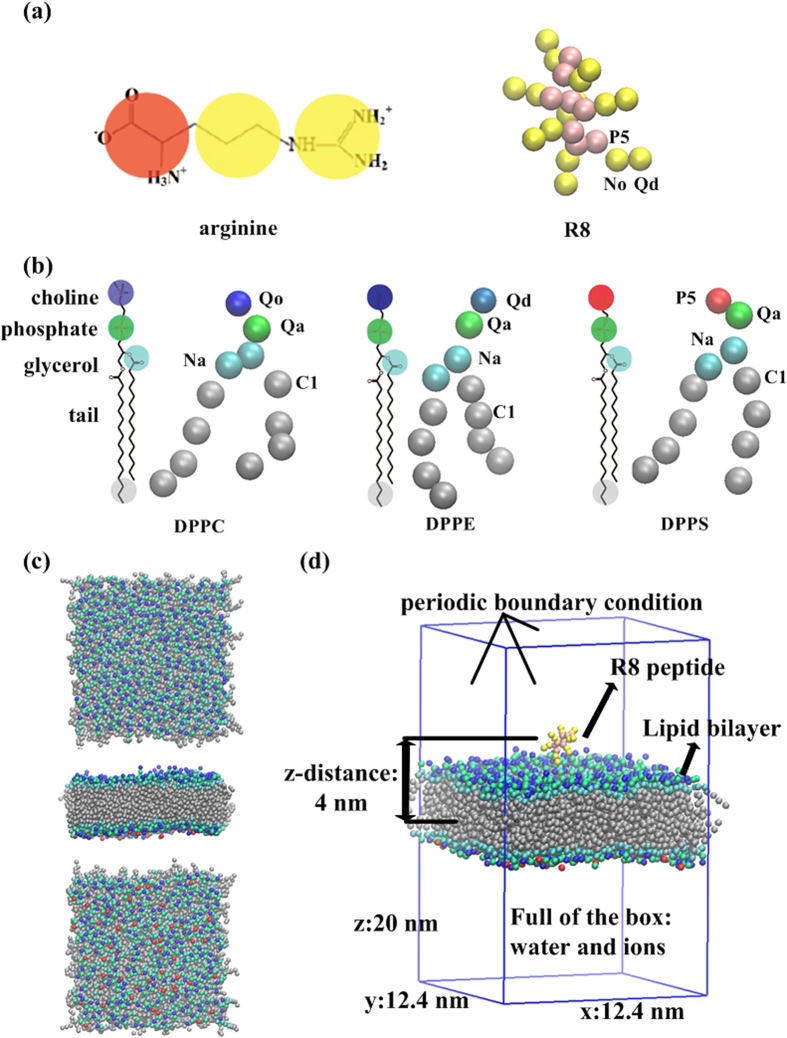
Structures of polyarginine (R8), lipid molecules, the asymmetric lipid bilayer, and the simulation box. Mapping of polyarginine R8 (**a**) and lipid molecules (**b**) in the framework of the Martini force field. (**c**) Top, side, and bottom views of the asymmetric lipid bilayer (area per lipid, 0.62 nm^2^). (**d**) Detailed information of the simulation box (membrane area per lipid, 0.62 nm^2^).

The polyarginine peptide (R8) model was built following the extended Martini force field for proteins (Martini_v2.1)^27^. In brief, the R8 peptide contains eight arginine amino acids with backbone bead (P5) and side chain beads (No and Qd) in each arginine amino acid (Fig. 1a), and each R8 peptide has eight positive charges. The bond, angle, and dihedral potential energy functions are used to simulate bonded interactions, whereas nonbonded interactions are represented by the L–J potential and Coulombic energy functions. The corresponding expressions and parameters are shown in Table S1.

This study used an asymmetric human erythrocyte membrane model^17^, which involves three lipid types, including dipalmitoyl phosphatidylcholine (DPPC), dipalmitoyl phosphatidylethanolamine (DPPE), and dipalmitoyl phosphatidylserine (DPPS). Mapping of lipid molecules based on the Martini CG force field (Martini_v2.1)^25^ is shown in Fig. 1b. Choline groups of lipids are represented by the CG beads Qo, Qd, and P5 for DPPC, DPPE, and DPPS, respectively. The phosphate groups of all lipids are represented by Qa. The glycerol groups and carbon tails of all lipids are represented by Na and C1, respectively. The expressions and parameters of bonded interactions (bond and angle potential) and nonbonded interactions (L-J potential and Coulombic energy) for lipid in the Martini CG force field^25^ are detailed in Table S2. In the outer leaflet of the membrane, the lipid ratio is 9:1 for DPPC and DPPE; in the inner membrane leaflet, the lipid ratio is 3:5:2 for DPPC, DPPE, and DPPS. The special lipid ratio was chosen on the basis of lipid composition of human erythrocyte membrane, following the study of Tian *et al.*^28^. In their study, seven different membranes were designed to investigate the influence of different lipids. Results showed that the presence of DPPE has no significant effect on the interaction and they mainly used the human erythrocyte membrane to indicate the endosomal escape mechanism. Up to 480 CG lipids are present in the asymmetric membrane. The lipid number was similar to the previous studies which were 512^29^ and 300^10^, respectively. When the membrane is tensionless, the area per lipid is 0.62 nm^2^ (Fig. 1c) which matches the experimental result^30^. The improved area per lipid was used to model the tension of the membrane. A tension was used to stretch the membrane in the xy-directions until the area per lipid achieved 0.65, 0.70, 0.75, and 0.80 nm^2^. The largest area per lipid of the tensed membrane was selected on the basis of the experimental results, which show that dehydrated lipid vesicles can bear a surface area increase of 28%^31^. Each membrane was equilibrated in the simulation system that contains lipids, water, and ions for 200 ns^20^.

Detailed information of the simulation box is provided in Fig. 1d. In the initial state, the center of mass separation distance in the z-direction between R8 and the membrane (z-distance) was 4 nm. Three initial distributions of R8 peptides (in-line, dispersed, and clustered) were investigated. The length and width of the simulation box in the xy-directions were dependent on the tension of the lipid bilayer. The height of the simulation box was fixed as 20 nm in the z-direction. After energy minimization, a force constant of 1000 KJ mol^−1^ nm^−2^ was used to constrain the lipid bilayer and peptides to equilibrate with water and ions for 200 ns. After the constrained simulations, long MD simulations of 1000 ns for each case were performed to ensure that the system is in equilibrium. The system temperature was kept at 323 K using a Berendsen thermostat^32^, and periodic boundary conditions were used in all simulations. The van der Waals interaction cutoff was 1.2 nm, and the L-J potential was smoothly shifted to zero from 0.9 nm to 1.2 nm to reduce cut-off noise. Particle mesh Ewald summation (PME) method^33^ was used to determine electrostatic interactions. Previous study^29^ has shown that with PME method, pore formation was observed in the case of PAMAM dendrimer-lipid bilayer interaction, which agreed well with experimental results. However, the Martini force field was parameterized with the cut-off method of electrostatic interaction. Therefore, to further assess the effect of PME method on the membrane properties, we compared properties of membranes with PME and cut-off methods, respectively (Table S3). The results show that the use of PME method brings no significant effect on the membrane properties. All simulations were performed by the GROMACS 4.5.4 package^34^, and results are represented by Visual Molecular Dynamics (VMD) 1.9 software^35^.

## Results and Discussion

### Effect of R8 concentration

**Figure 2:**
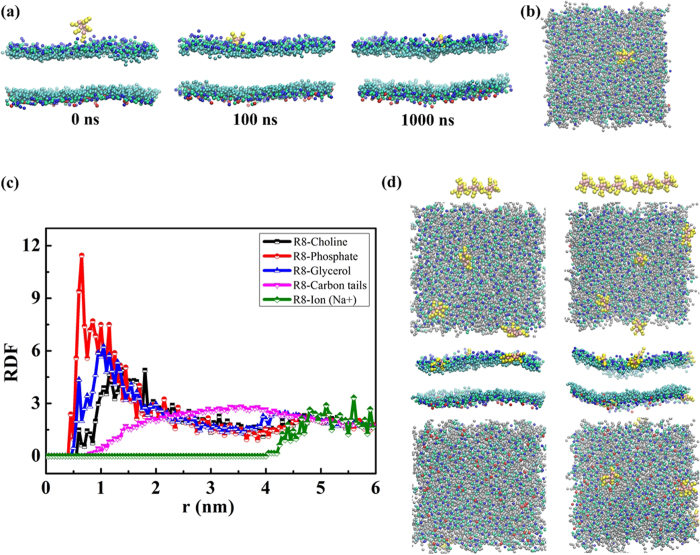
Results of interactions between R8 peptides and the asymmetric membrane without tension: Side view of snapshots of R8-asymmetric membrane (area per lipid 0.62 nm^2^) interactions at 0 ns (**a**), 100 ns, 1000 ns, and top view of snapshot at 1000 ns (**b**). (**c**) RDFs of lipid groups and ions with respect to the R8 peptide. (**d**) Snapshots of R8–membrane interactions with different R8 peptide concentrations in the equilibrium state. From top to bottom: Initial distributions of three and six peptides. Top, side, and bottom views of snapshots of R8–asymmetric membrane interactions.

To obtain insights into the mechanism of R8-biomembrane interactions, we simulated the interactions between an asymmetric membrane without tension and R8 peptides with different peptide concentrations (Fig. 2). The peptide and lipid ratio was used to indicate the peptide concentration (one, three, or six R8 peptides to 480 lipids). Results show that one R8 peptide is rapidly absorbed at the outer leaflet of the membrane without penetration in the equilibrium state at low concentrations (one R8 to 480 lipids) (Fig. 2a,b) because of the presence of a high-energy barrier for one peptide that penetrates through the membrane. This phenomenon has also been illustrated in a recent study which investigated the potential of mean force (PMF) of interactions between single R9 and asymmetric membranes^10^.

To understand the observed interactions further, we analyzed the radial distribution functions of lipid groups and ions with respect to the R8 peptide (Fig. 2c). The highest peak is observed in the lipid phosphate groups (**red line in** Fig. 2c), the second-highest peak is observed in the glycerol groups (**blue line in** Fig. 2c), and the lowest peak is observed in choline groups (**black line in** Fig. 2c). These results indicate that R8 peptides are adsorbed at the bottom of lipid head groups and not on the lipid surface. Such a finding is attributed to the fact that the positively charged R8 peptide repulses positively charged choline groups and ions and attracts negatively charged phosphate groups. A similar phenomenon has also been observed in a previous study where peptides accumulated in the outer membrane leaflet and localized between the phosphate groups and carbon chains of lipids^2^.

To investigate the case of high R8 concentration, we increased the R8 peptide number to three and six (Fig. 2d). Results show that R8 peptides remain absorbed at the bottom of lipid head groups with three R8. However, two R8 peptides can penetrate through the membrane in the case of concentration with six R8 peptides.

The phenomena observed under high R8 concentration may be explained in two aspects. The first involves enhanced electrostatic interactions in the system. A recent study has shown that electrostatic interactions play a major role in the interactions between cationic nanoparticles and negatively charged biomembrane^28^. Higher concentrations of R8 peptides yield more positive charges in the system, thereby enhancing the electrostatic attraction between peptides and DPPS lipids in the inner membrane leaflet. To verify the importance of electrostatic attraction between peptides and DPPS lipids in the inner membrane leaflet, we also simulated the interactions in a symmetric membrane (Fig. S1). The DPPS percentage is 10% in the inner leaflet of the asymmetric membrane and 10% in each leaflet of the symmetric membrane (Fig. S1a). These results are dramatically different compared with the case of the asymmetrical biomembrane. Snapshots of R8–symmetric membrane interactions in the equilibrium state (Fig. S1b) show that all six R8 peptides are adsorbed at the bottom of lipid head groups. No penetration is observed when negatively charged DPPS lipids appear at the outer membrane leaflet.

**Figure 3:**
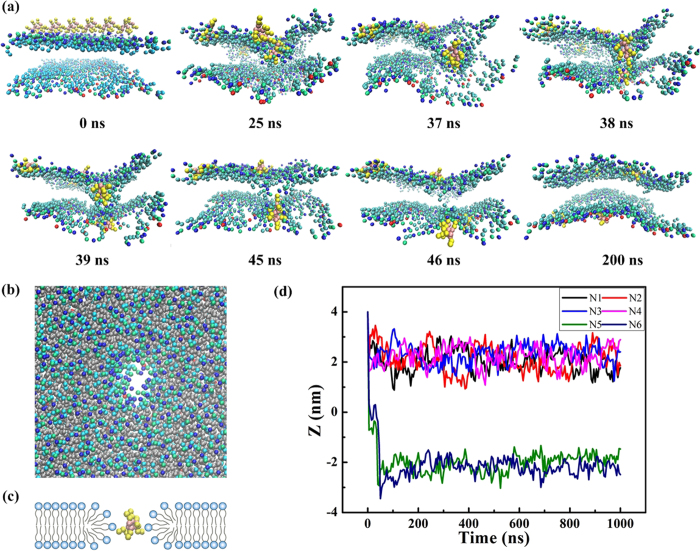
Penetration and mechanism of R8 peptides in six R8–asymmetric membrane interactions: (**a**) Time sequence of snapshots of interactions between six R8 peptides and the asymmetric membrane (area per lipid, 0.62 nm^2^). (**b**) Top view of the membrane at 38 ns. For clarity, the peptides are not shown. (**c**) Schematic of the peptide penetration mechanism. (**d**) Time sequence of the center of mass separation distance in the z direction (z-distance) between six peptides and the membrane.

The second reason involves the cooperation effect of peptides. Under enhanced electrostatic interactions, the cooperation effect of peptides can promote penetration. A previous study showed the cooperation effect of dendrimers during insertion into a bilayer^26^. Multi-dendrimers can induce formation of membrane curves, and these membrane changes positively affect dendrimers located in curved regions for insertion into the membrane. The cooperation effect of multi-R8 peptides also follows this phenomenon, and the detailed penetration process of the six R8 peptides is shown in Fig. 3. Based on the results described above, we can infer that an R8 peptide with low concentration (one R8 to 480 lipids) cannot penetrate the membrane because of its high energy barrier. However, R8 peptides with a high concentration (six R8 to 480 lipids) can penetrate the membrane because of enhanced electrostatic interactions and the cooperation effect of peptides.

### Effect of membrane tension

**Figure 4:**
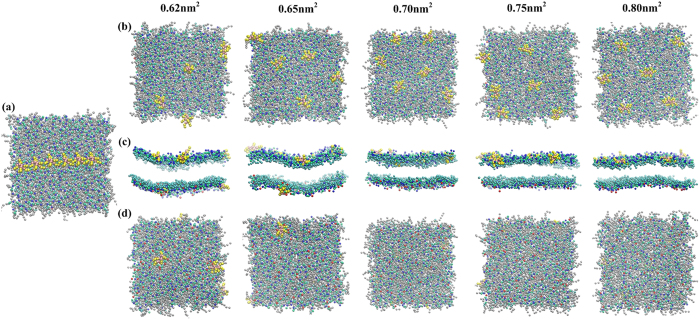
Snapshots of interactions between six R8 peptides and different tensed asymmetric membranes in the equilibrium state: (**a**) Initial distribution; top view (**b**), side view (**c**), and bottom view (**d**) of snapshots of six R8-tensed membrane interactions. Area per lipid (0.62, 0.65, 0.70, 0.75, and 0.80 nm^2^) is used to denote membrane tension.

To understand the effect of membrane tension on the R8–biomembrane interactions with penetration, we simulated the interactions between six R8 peptides and tensed asymmetric membranes with areas per lipid of 0.65, 0.70, 0.75, and 0.80 nm^2^ (Fig. 4). The interaction observed in a tensionless membrane with an area per lipid of 0.62 nm^2^ was used as a reference. In the case of the tensionless membrane, two of six R8 peptides can penetrate through the membrane from the outer leaflet to the inner leaflet and adsorb onto the inner membrane leaflet (Fig. 4). The time required for two R8 peptides to arrive at the inner membrane leaflet is 50 ns. When the area per lipid increases to 0.65 nm^2^, only one of six R8 peptides can pass through the membrane. In addition, 760 ns is required for one R8 peptide to arrive at the inner membrane leaflet. This time period increases with increasing membrane tension. When the area per lipid further increases to 0.70, 0.75, and 0.80 nm^2^, all of the R8 peptides are blocked at the outer leaflet of the membrane. These results indicate that peptide penetration can be restrained with increasing membrane tension.

The results described above are different from conventional understanding. It is generally thought that tension causes disorder in the structure of membranes and reduces their strength, which facilitates penetration. For instance, interactions between large molecules (charged dendrimer) and a DPPC symmetric membrane with tension have been simulated by using the dissipative particle dynamics method. Permeability can be enhanced by increasing membrane tension because sparse lipids in the lipid-poor region provide space through which the dendrimer can penetrate^14^. However, for the small molecules such as peptide R8, our simulation results found that the penetration of R8 peptides can be restrained by membrane tension. This interesting result will be explained in the following analysis.

To understand the effect of membrane tension on peptide interactions, we first investigated the translocation mechanism of R8 peptides by tracking the penetration process of six R8 peptides in an asymmetric and tensionless membrane with area per lipid of 0.62 nm^2^ (Fig. 3). Two R8 peptides induce the downward curvature of the membrane at 25 ns, and one of the R8 peptides in the lower location interacts with lipids in the inner leaflet of the membrane at 37 ns (Fig. 3a). Then, a hole forms in the membrane at 38 ns, and two R8 peptides penetrate along this channel. After 39 ns, one R8 peptide penetrates the membrane thoroughly and the other R8 peptide arrives at the inner leaflet at around 46 ns. Figure 3b shows the top view of the membrane hole at 38 ns (for clarity, the R8 peptides are not shown). Combining the top and side views of the membrane hole, we can infer that the hole is hydrophilic, which indicates that lipids around the hole turn to ensure that lipid tails do not interact with water molecules (Fig. 3c). The peptides penetrate through the membrane by inducing hydrophilic holes in it. Accompanied by the arrival of the inner membrane leaflet of R8 peptides, the hole disappears at around 50 ns. After 200 ns, the system is in the equilibrium state, and two of six R8 peptides can penetrate through the membrane. The quantified z-distance can also illustrate that four R8 peptides remain in the lipid head group of the outer leaflet while two R8 peptides adsorb onto the inner leaflet (Fig. 3d).

**Figure 5:**
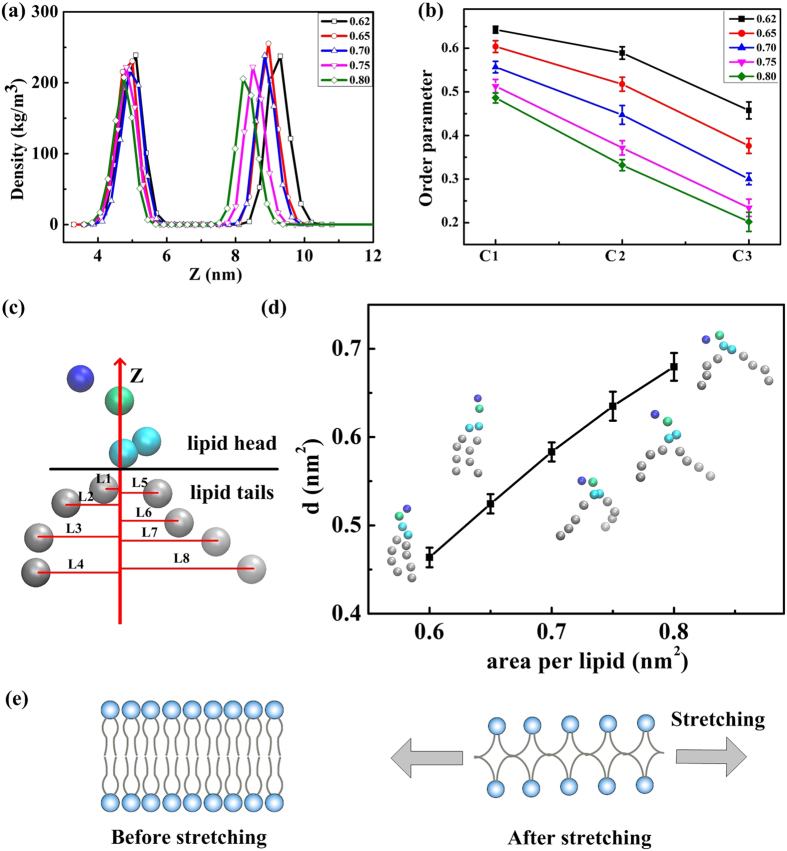
Structural properties of the tensed asymmetric membrane: (**a**) Densities of lipid choline groups of different tensed membranes. (**b**) Order parameters of lipid tails in six R8–tensed membrane interactions. (**c**) Schematic of the average square distance between lipid tails and the middle z-axis. (**d**) Average square distance between lipid tails and the middle z-axis of different tensed membranes. Snapshots of individual lipids in the different tensed membranes are included in the figure. (**e**) Schematic of membrane stretching.

In the following discussion, we analyze the structural properties of the tensed asymmetric membrane to investigate the effect of membrane tension on the interactions. These properties include membrane thickness, order parameter of lipid tails, and potential of one lipid tail intertwining with others (Fig. 5). Figure 5a shows the densities of lipid choline groups in different tensed membranes. The distance between the two peaks of each line represents the membrane thickness. Thicknesses of 4.2, 4.1, 3.94, 3.675, and 3.46 nm are observed for membranes with areas per lipid of 0.62, 0.65, 0.70, 0.75, and 0.80 nm^2^, respectively. These results indicate that the membrane becomes thinner with increasing membrane tension.

To investigate lipid properties, the order parameter of lipid tails in membranes with different tensions was analyzed in Fig. 5b. The order parameter was calculated using Eq. 1:


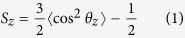


where *θ*_*z*_ is the angle between the z-axis of the simulation box and the vector from beads C_n+1_ to C_n−1_. The order parameter was calculated by averaging all lipid tails and over last 200 ns in the simulations. Results show that the order parameter decreases with increasing membrane tension.

We observed from the snapshots of individual lipids (Fig. 5d) that the distance between two tails of each individual lipid in the xy-plane increases with increasing membrane tension. This result indicates that the tails can easily intertwine with other tails nearby. To quantify the potential of one lipid tail intertwining with tails of other lipids, we defined a parameter *d* for the lipid named as “average square distance between tails and middle z-axis” (Fig. 5c). This parameter *d* represents the average deviation degree of carbon tails to the z-axis. The middle z-axis is defined as the z-axis passing through bead Qa, as shown in Fig. 1(b). For individual lipids, the equation of average square distance is (Eq. 2)


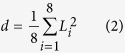


where *L*_*i*_ is the distance between the tail bead C1 and z-axis. To obtain the statistic average square distance, we calculated the average *d* of all 480 lipids in the membrane and over the last 25 ns using the VMD tool command language console. Figure 5d shows that the average square distances (*d*) are 0.466, 0.519, 0.587, 0.637, and 0.679 nm^2^ for tensed membranes with areas per lipid of 0.62, 0.65, 0.70, 0.75, and 0.80 nm^2^, respectively. These results indicate that the potential of lipid tails intertwining together increases with increasing membrane tension. Figure 5e shows the schematic of membrane changes under stretching.

The above results can be validated by the experimental study of Sakamoto *et al.*^36^. They used critical packing parameter (CPP) to determine the lipid topology. CPP = *v*/(*a* × *l*), where *v* is the volume of the hydrophobic part; *a* is the area per lipid at the hydrophilic-hydrophobic interface; *l* is the hydrophobic part length. If CPP > 1, the membrane has a positive curvature; if CPP < 1, the membrane has a negative curvature. Their experiments showed that the penetration of R8 peptides into the erythrocyte is enhanced in the hypotonic condition (positive curvature) while is resistant in the hypertonic condition (negative curvature). Their results can provide a good support for our claim the lipid topology plays an important role in the R8 peptide-biomembrane interactions.

Standard Martini water model was used in this work. However, the polarized Martini water model has been developed, which performs interactions between charged and polar groups in a low-dielectric medium more realistically^37^. For instance, the electroporation of a lipid bilayer with polarized water model is in good agreement with atomistic simulation results. To further investigate the effect of water model on the peptide penetration, we performed simulations of interactions between six R8 peptides and membranes (area per lipid 0.62 and 0.8 nm2) with polarized water model (Fig. S2). The results show that two of six R8 peptides penetrate through the membrane (area per lipid 0.62 nm2) while no peptide penetrates through the membrane (area per lipid 0.8 nm2), which are as same as the cases with non-polarized water model.

All simulations in this work are under the framework of Martini coarse-grained force field. As any other models, Martini has its limitations^38^. Compared with atomistic force fields that have been tested for several years, Martini coarse-grained force field has limited chemical and spatial resolution. Moreover, Martini model has been proved to break down in the case of pore formation in the lipid bilayer. Bennett and Tieleman have shown that pore formation and membrane structure defect are observed in the atomistic simulation, however the structure (without pore formation) is very different from the Martini model in their study^39^. However, Martini force field has a longer time (millisecond) and length (micrometer) scales than atomistic method, which can simulate biological phenomena such as nanoparticle translocation process. Therefore, in spite of limitations mentioned above, Martini model is suitable for simulating nanoparticle-biomembrane interactions as the case in this study.

## Conclusions

CGMD simulations were performed to investigate interactions between the R8 peptide and membranes under tension. R8 peptides with low concentration (one R8 to 480 lipids) cannot penetrate the tensionless membrane because of its high energy barrier. However, R8 peptides with high concentration (six R8 to 480 lipids) can penetrate the membrane because of enhancements in electrostatic interactions and the cooperation effect of peptides. Membrane tension can restrain peptide penetration. With increasing membrane tension, the membrane becomes thin, lipid tails are more disordered, and the potential of intertwining between neighboring lipid tails increases. The lipid topology such as lipid tail order parameter and potential of intertwining between neighboring lipid tails can play an important role in the R8 peptide-biomembrane interactions. These results provide molecular insights into the translocation mechanism of cell-penetrating peptides, suggestions for nanomedical designs, and applications of therapeutic purposes for drug and gene delivery.

## Additional Information

**How to cite this article**: He, X.C. *et al.* Coarse-grained molecular dynamics studies of the translocation mechanism of polyarginines across asymmetric membrane under tension. *Sci. Rep.* **5**, 12808; doi: 10.1038/srep12808 (2015).

## Supplementary Material

Supplementary Information
