# Supplementary material for: Coarse-grained molecular dynamics studies of the translocation mechanism of polyarginines across asymmetric membrane under tension
Source: Sci Rep. 2015 Aug 3;5:12808. doi: 10.1038/srep12808 (PMC4522684; doi:10.1038/srep12808)
Supplement: Supplementary Information [file srep12808-s1.doc]

**Supplemental information**

**Coarse-grained molecular dynamics studies of the translocation mechanism of polyarginines across asymmetric membrane under tension**

XiaoCong He^a,b^, Min Lin^b,c^, BaoYong Sha^b,d^, ShangSheng Feng^b^, XingHua Shi^e^, ZhiGuo Qu^a,b#^, Feng Xu^b,c#^

*^a^* *Key Laboratory of Thermo-Fluid Science and Engineering of Ministry of Education, School of Energy and Power Engineering, Xi’an Jiaotong University, Xi’an 710049, P.R. China*

*^b^ Bioinspired Engineering and Biomechanics Center (BEBC), Xi’an Jiaotong University, Xi’an 710049, P.R. China*

*^c^ The Key Laboratory of Biomedical Information Engineering of Ministry of Education, School of Life Science and Technology, Xi’an Jiaotong University, Xi’an 710049, P.R. China*

*^d^ Institute of Basic Medical Science, Xi’an Medical University, Xi’an 710021, P.R. China*

^e^ The State Key Laboratory of Nonlinear Mechanics, Institute of Mechanics, Chinese Academy of Sciences

*^#^ Corresponding authors:* [*zgqu@mail.xjtu.edu.cn*](mailto:zgqu@mail.xjtu.edu.cn)*,* [*fengxu@mail.xjtu.edu.cn*](mailto:fengxu@mail.xjtu.edu.cn)

**
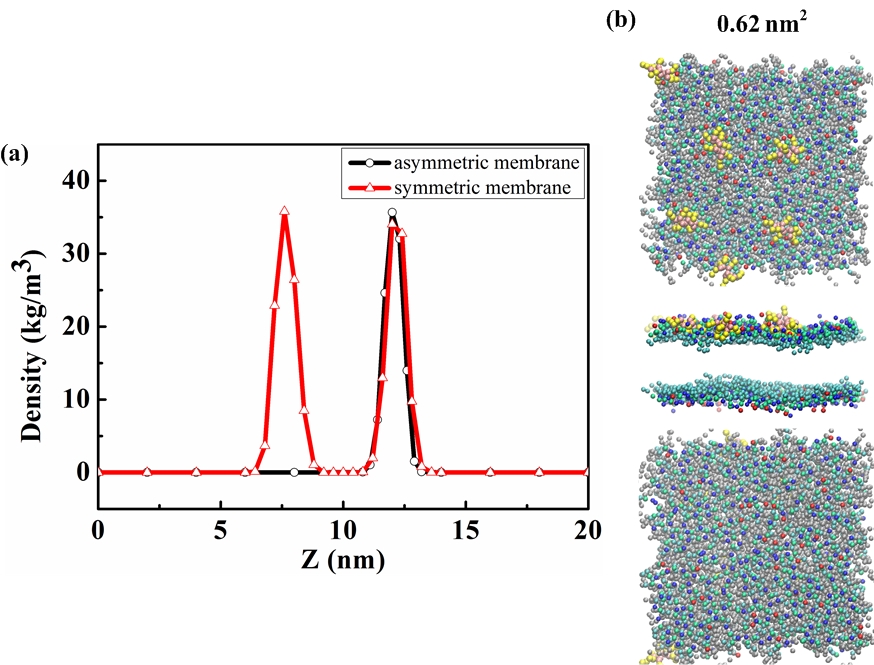
**

**Supplemental Figure 1. Results of six R8–symmetric membrane interactions in the equilibrium state**: (a) Densities of DPPS lipid choline groups in asymmetric and symmetric membranes. (b) Snapshots of six R8–symmetric membrane interactions in the equilibrium state. From top to bottom: top, side, and bottom views.


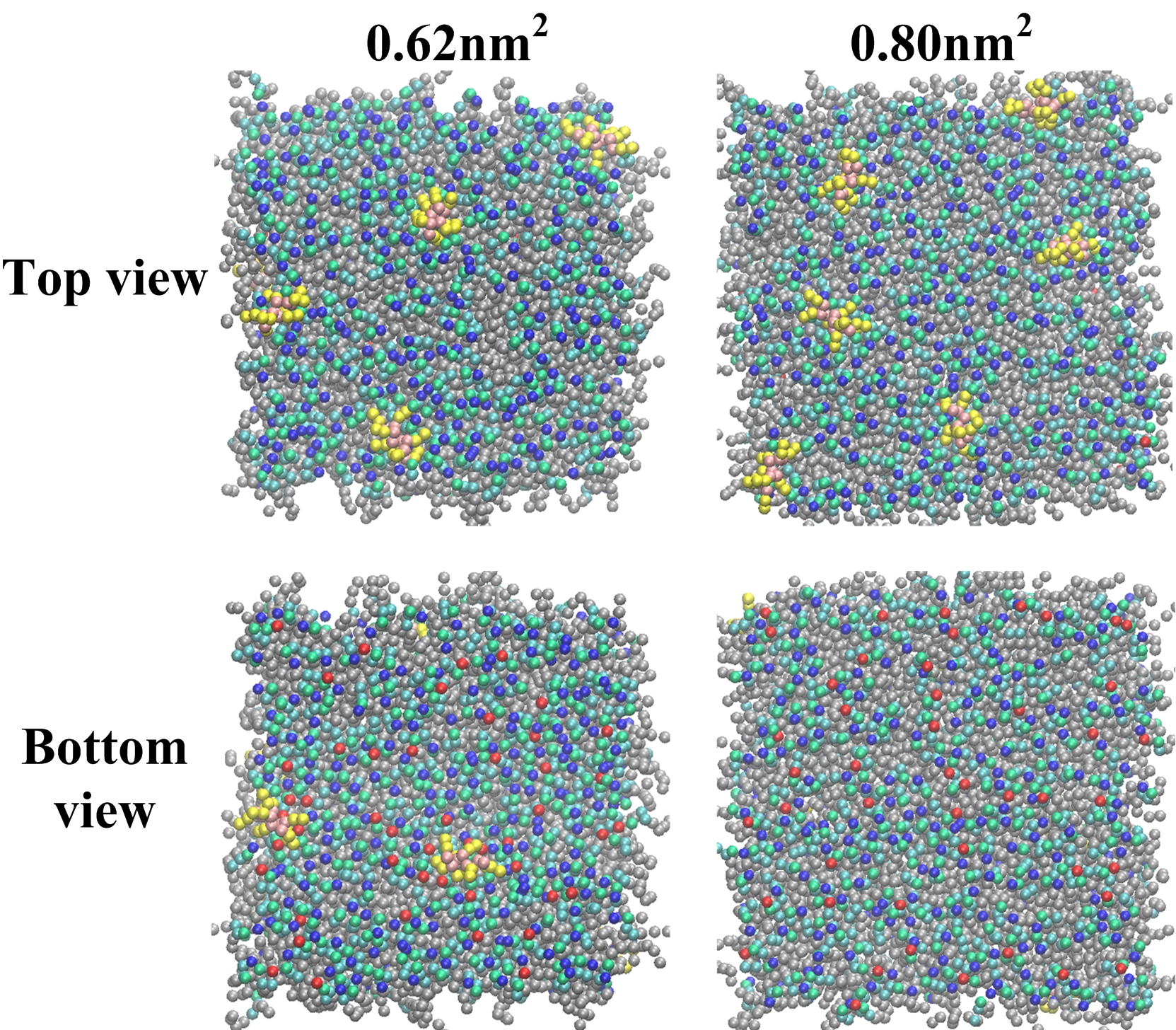


**Supplemental Figure 2. Snapshots of six R8 peptides–asymmetric membrane interactions in the equilibrium state with polarized water model (top and bottom views)**. The areas per lipid are 0.62 and 0.8 nm^2^, respectively.

**Supplemental Table 1.** Detailed description of the R8 peptide model in the Martini CG force field

| **Interaction** | **Type** | **Equation** | **Parameter** | |
| --- | --- | --- | --- | --- |
| Nonbonded interactions | L-J potential | $U_{LJ}(r)=4\varepsilon_{ij}\left[(\frac{\sigma_{ij}}{r})^{12}-(\frac{\sigma_{ij}}{r})^{6}\right]$ | $\sigma_{ij}$=0.47 nm | |
|  | Coulombic energy | $U_{el}(r)=\frac{q_{i}q_{j}}{4\pi\varepsilon_{0}\varepsilon_{r}r}$ | $\varepsilon_{r}$=15 | |
| Bonded interactions | Bond, angle and dihedral potential energy  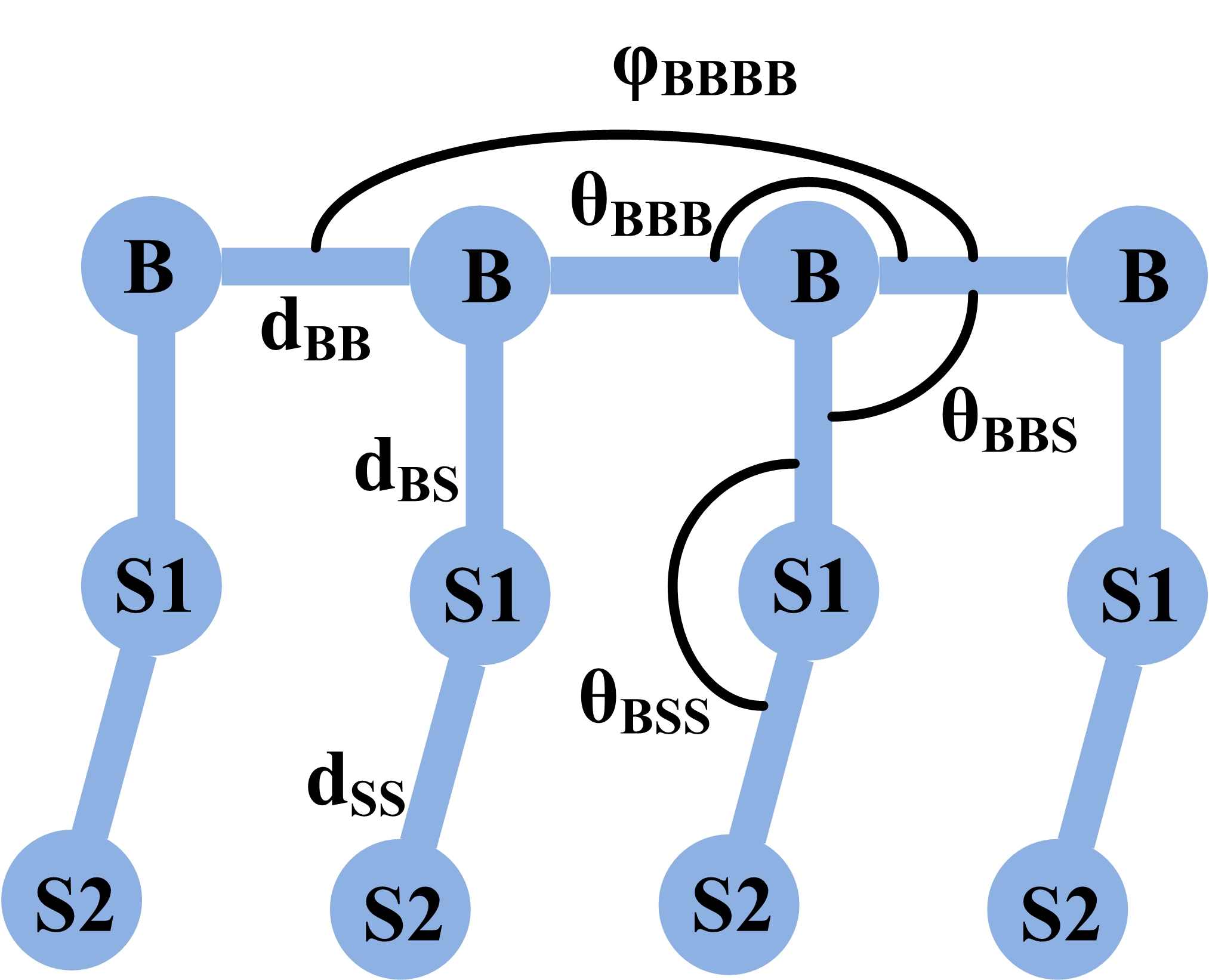  Schematic of bond, angle, and dihedral of polyarginine. (B: backbone bead; S: side chain bead) | $V_{b}=\frac{1}{2}K_{b}(d_{ij}-d_{b})^{2}$ | $d_{BB}$=0.35 nm | $K_{BB}$=1250 KJ mol^−1^ nm^−2^ |
|  |  |  | $d_{BS}$=0.33 nm | $K_{BS}$=5000 KJ mol^−1^ nm^−2^ |
|  |  |  | $d_{SS}$=0.33 nm | $K_{SS}$=5000 KJ mol^−1^ nm^−2^ |
|  |  | $V_{a}=\frac{1}{2}K_{a}\left[\cos(\theta_{ijk})-\cos(\theta_{a})\right]^{2}$ | $\theta_{BBB}$=96° | $K_{BBB}$=700 KJ mol^−1^ |
|  |  |  | $\theta_{BBS}$=100° | $K_{BBS}$=25 KJ mol^−1^ |
|  |  |  | $\theta_{BSS}$=180° | $K_{BSS}$=25 KJ mol^−1^ |
|  |  |  | =60° | =400 KJ mol^−1^ |

**Supplemental Table 2.** Detailed description of the lipid model in the Martini CG force field

| **Interaction** | **Type** | **Equation** | **Parameter** | |
| --- | --- | --- | --- | --- |
| Nonbonded interactions | L-J potential | $U_{LJ}(r)=4\varepsilon_{ij}\left[(\frac{\sigma_{ij}}{r})^{12}-(\frac{\sigma_{ij}}{r})^{6}\right]$ | $\sigma_{ij}$=0.47 nm | |
|  | Coulombic energy | $U_{el}(r)=\frac{q_{i}q_{j}}{4\pi\varepsilon_{0}\varepsilon_{r}r}$ | $\varepsilon_{r}$=15 | |
| Bonded interactions | Bond potential energy | $V_{b}=\frac{1}{2}K_{b}(d_{ij}-d_{b})^{2}$ | $R_{bond}$=0.47 nm | $K_{bond}$=1250 KJ mol^−1^ nm^−2^ |
|  | Angle potential energy | $V_{a}=\frac{1}{2}K_{a}\left[\cos(\theta_{ijk})-\cos(\theta_{a})\right]^{2}$ | $\theta_{0}$=180° | $K_{angle}$=150 KJ mol^−1^ rad^−2^ |

**Supplemental Table 3.** Comparison of properties of membrane with PME or cut-off methods. Each membrane was equilibrated in the simulation system that contains lipids, water, and ions for 600 ns. The treatment of electrostatic interaction used PME and cut-off methods, respectively.

|  | **Are per lipid**  **(nm^2^)** | **Thickness (nm)** | **Lipid tail order parameter** | | |
| --- | --- | --- | --- | --- | --- |
|  |  |  | C1 | C2 | C3 |
| **Membrane with cut-off** | 0.613 | 4.26 | 0.6438 | 0.5829 | 0.4573 |
| **Membrane with PME** | 0.615 | 4.2 | 0.6487 | 0.5866 | 0.4526 |
